# Evaluation of the Iatrogenic Sciatic Nerve Injury following Double Pelvic Osteotomy Performed with Piezoelectric Cutting Tool in Dogs

**DOI:** 10.3390/vetsci9060259

**Published:** 2022-05-29

**Authors:** Roberto Properzi, Francesco Collivignarelli, Andrea Paolini, Amanda Bianchi, Massimo Vignoli, Ilaria Falerno, Andrea De Bonis, Roberto Tamburro

**Affiliations:** 1 Clinica Veterinaria Properzi, Via Santa Maria del Campo 16, 16035 Rapallo, Italy; dr.properzi@libero.it; 2 Faculty of Veterinary Medicine, Veterinary Teaching Hospital, University of Teramo, 64100 Teramo, Italy; fcollivignarelli@unite.it (F.C.); Bianchi.amandal@gmail.com (A.B.); ifalerno@unite.it (I.F.); adebonis@unite.it (A.D.B.); rtamburro@unite.it (R.T.); 3 Clinica Veterinaria San Marco, 35030 Veggiano, Italy

**Keywords:** double pelvic osteotomy, hip dysplasia, piezoelectric cutting tool, sciatic nerve, dog

## Abstract

(1) Background: The double pelvic osteotomy (DPO) is a prophylactic surgical procedure associated with 0.4% incidence of sciatic nerve injury. The piezoelectric cutting tool is a surgical device able to involve only mineralized tissue avoiding neurovascular tissue and other soft tissue. This study aimed to evaluate the sciatic nerve injury observed in dogs underwent iliac osteotomy performed using the piezoelectric cutting tool. (2) Methods: Dogs underwent DPO performed with piezoelectric cutting tool were included. Neurological assessment was performed 6 and 24 h after surgery and then repeated 12 days, 4 and 8 weeks after surgery. Temporary and or permanent sciatic nerve injury were recorded. (3) Results: 84 DPOs performed in fifty dogs were included. No temporary/permanent neurological disease associated with iatrogenic damage of the sciatic nerve were observed. (4) Conclusions: The iliac osteotomy performed with piezoelectric cutting tool was not associated to iatrogenic sciatic nerve injury.

## 1. Introduction

The double pelvic osteotomy (DPO) is a corrective surgical procedure performed in dogs affected by hip dysplasia [1,2]. The surgical plan includes a pubic ostectomy followed by an ipsilateral iliac osteotomy. The consequent acetabular ventroversion allows to increase the femoral head coverage. A preangled plate is used to fix the ilial osteotomy [1,2,3,4,5,6,7].

Ideal DPO candidates include dogs from 4.5 to 9 months of age with evidence of hip joint dorsal subluxation, minimal osteoarthritic changes, minimal acetabular filling, subluxation angle ≤ 25 degree and distraction index up to 1° [1]. Recently double pelvic osteotomy was successfully performed in eight dogs older than 9 months of age [2].

A paper focused on the DPO complications reported that injury of the sciatic nerve occurred in 0.4% in a study population of 305 dogs and 458 DPOs [8].

Despite the sciatic nerve injury complication rate following DPO being low, it has always been considered a feared complication. The deep location associated with proximity to the pelvic bone and limited visibility make the sciatic nerve vulnerable to damage during the iliac osteotomy.

Following DPO, the osteotomy is generally performed using an oscillating saw and then may be completed using an osteotome [1,3]. Despite the iliac bone is always protected using Hohmann and/or Gelpi retractors, iatrogenic sciatic nerve injury may occur. Nerve injuries are classified as class I (neurapraxia), class II (axonotmesis) and class III (neurotmesis) [9]. Neurapraxia refers to nerve conduction failure because of disruption of myelin, causing structural changes and partial loss of axonal continuity. Neurologic signs include conscious proprioceptive sensory deficits, scuffing of the dorsal aspect of the 3rd and 4th digits and decreased flexor withdrawal of the hock joint. A full quick recovery is generally observed within 10–15 days [9,10]. In case of axonotmesis some or all of the axons of the nerve are damaged without involvement of the connective tissue support that remains intact. These axons can regrow along the connective tissue scaffold. Substantial motor, proprioceptive and nociceptive dysfunction is expected with this type of injury, the extent of which depends on the number of axons damaged. Neurogenic muscle atrophy is likely with this class of injury. In case of neurotmesis, complete disruption of the axons of the nerve and connective tissue support. These axons will not regrow without surgery. Complete motor, proprioceptive and nociceptive dysfunction occurs with this class of injury. Neurogenic muscle atrophy is to be expected. Class II and may be clinically indistinguishable from a class III injury [9,10,11].

In order to mitigate the iatrogenic sciatic nerve injury great interest is associated to device able to produce a safer cut.

**Figure 1:**
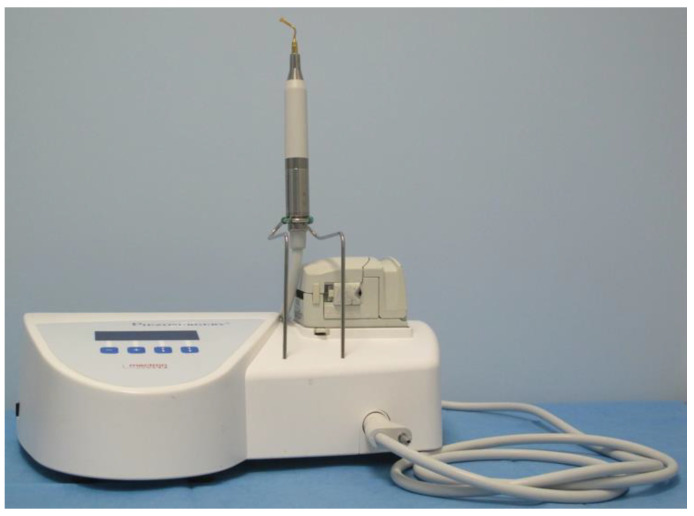
Piezoelectric cutting tool device (Mectron S.P.A., Carasco-Genova, Italy).

The Piezoelectric devices use low frequency ultrasonic waves (25–30 kHz) that are created by the piezoelectric effect. The applied power can be modulated between 2.8 and 16 W producing linear micro-vibrations between 60 and 210 μm and the machine is programmable in accordance with the density of the bone being cut [12] (Figure 1). The micro-movements created within the chosen frequency range is able to involve only mineralized tissue avoiding neurovascular tissue and other soft tissue. Piezoelectric cutting devices were first used in oral and neurosurgery [13,14,15,16,17,18]. Piezoelectric osteotomy has also been associated to a proper bone healing [19,20,21].

According to the reference above we hypothesized that the iliac osteotomy may be performed without evidence of sciatic nerve injury using the piezoelectric cutting tool device.

For this reason the aim of this study is to retrospectively evaluate sciatic nerve injury observed in dogs underwent double pelvic osteotomy with the piezoelectric cutting tool device.

## 2. Materials and Methods

The study protocol was in accordance with institutional guidelines for research on animals and was approved by the Ethics Committee of the University of Teramo (Prot.n. 6125). All dog owners were fully informed about the procedures, and written informed consent was obtained.

Inclusion criteria were dogs who underwent unilateral DPO, single stage bilateral procedures and double stage bilateral DPOs in which iliac osteotomy was performed with the piezoelectric cutting tool. For each dog orthopedic examination, preoperative hip X-rays, DPO surgery followed by postoperatively radiographic evaluation were performed. Follow-ups were carried out at 12 days, 4 and 8 weeks after surgery.

Each dog was sedated with morphine (0.2 mg/kg IM, Morfina Cloridrato, Aic Atc, Roma, Italy) and acepromazine (0.05 mg/kg IM, Prequillan ATI, Bologne, Italy) 20 min before induction. After preoxygenation for 5 min, anesthesia was induced with propofol (4 mg/kg IV, Propovet, Zoetis, Roma, Italy). Cefazoline (20 mg/kg, IV Cefazolina, Teva, Milan, Italy) was administered at induction and every 90 min throughout the procedure. After orotracheal intubation, anesthesia was achieved with sevoflurane and oxygen. No epidural anesthesia was performed.

Surgical approach was performed according to the pubic and iliac bones were approached according to the reference points proposed by Slocum [22]. Each dog was placed in lateral recumbency. The upper leg was abducted and slightly extended by the assistant, in order to visualize the pectineus muscle. A 4–5 cm incision was made over the origin of the pectineus muscle and its pubic origin was elevated from the iliopectineal eminence of the pubis in order to identify the pubic bone. A curved retractor was used to protect the obturator nerve. The pubic ostectomy (1 cm) was performed by a rongeur bite, and the sacrotuberous ligament was not released [2]. The subcutaneous tissue and skin were routinely closed. Then the leg was returned in normal position allowing a standard lateral approach to the ilium. The origin of the deep gluteal muscle was elevated from the ilium using a large Hohmann and/or Gelpi retractors. The iliac osteotomy was performed from just caudal the sacro-iliac joint bone perpendicular to the long axis of the ilium in latero-medial direction. The osteotomy was performed using the piezoelectric cutting tool set at frequency of 29 kHz. The complete osteotomy was checked with an osteotome. The caudal segment was laterally rotated with a bone-holding forceps associated with a rotation level bar and then secured with a preangled plate. The iliac osteotomy was randomly fixed using plates from two manufactures. Surgical approach was routinely closed.

Post-operative laterolateral and ventrodorsal radiographic views were performed to inspect implant position.

Post-operative medications included Amoxicilline/Clavulanic acid (20 mg/kg q12 Amoxicillina/Acido Clavulanico Teva, Milan, Italy), Meloxicam (0.1 mg/kg q24, Metacam, Boehringer-Ingheleim, Milan, Italy) and Methadone (0.15 mg/kg q4 Senfortan, Decra, Pomigliano d’Arco, Naples, Italy) followed by Tramadol (4 mg/kg q8, Altadol, Formevet srl, Milan, Italy).

Each dog underwent a neurological assessment 6 and 24 h after surgery, and then repeated 12 days, 4 and 8 weeks after surgery. The examination was divided into the following parts: observation, palpation, examination of postural reactions, spinal reflexes and sensory evaluation. Nerve injuries were classified as followed: class I (neurapraxia), class II (axonotmesis) and class III (neurotmesis).

Evidence of sciatic nerve injury was considered in case of dropped-hock and knuckling of the digits, decreased withdrawal reflex and decreased/absent deep pain reaction. Full recovery was defined as no proprioceptive deficits, normal deep sensory perception upon squeezing digit 5, normal hock flexion on withdrawal. Complications regarding the sciatic nerve injury has been recorded as temporary or permanent injury. Further complications have been recorded but not analyzed and were classified as major/minor if a revision surgery was performed (major) or not (minor).

The DPO bone-healing was radiographically classified 2 months after surgery according to the following score: grade 0—no radiographic signs of healing, grade 1—incomplete osseous bridging and grade 2—complete osseous bridging [2].

All patients were discharged the day after the surgery dressing a E-collar 24/7 until the skin suture removal. A self-adhering bandage was applied on the iliac surgical wound. All dogs received Amoxicillin Clavulanate (20 mg/kg, q12 Amoxicillina/Acido Clavulanico, Teva, Milan, Italy) for seven days, Meloxicam (0.1 mg/kg, q24, Metacam, Boehringer-Ingheleim, Milan, Italy) for seven days. The owners were advised to keep their dog in the house for eight weeks, during which lead walking was allowed for 15 min three times a day.

## 3. Results

Overall, a total of 84 DPOs performed in fifty dogs were included. Of these, there were 28 males and 22 females. Presenting breeds included mixed large breed dog (9), German Shepherd (8), Border Collie (5), Golden Retriever (5), Labrador Retriever (5), Irish Setter (4), Rottweiler (3), Floated Clot Retriever (3), Bernese Mountain Dog (2), Dogue de Bordeaux (1), Argentine Dogo (1) and Saint Bernard Dog (1).

Body weight was 28.8 kgs ± 5.30 (mean ± ds) (range 15–38 kgs), age was 7 month-old ± 0.97 (mean ± ds) (range 5–8 month-old). Sixteen unilateral DPOs, thirteen single stage bilateral procedures (26) and twenty-one double stage bilateral procedures (42) were performed. In case of two-staged bilateral procedure, the controlateral DPO procedure was performed eight weeks after the previous one.

Double pelvic osteotomy plates from two different manufacturers were used: Depuy Synthes (*n* = 30) and Fixin DPO plates (*n* = 54)

**Figure 2:**
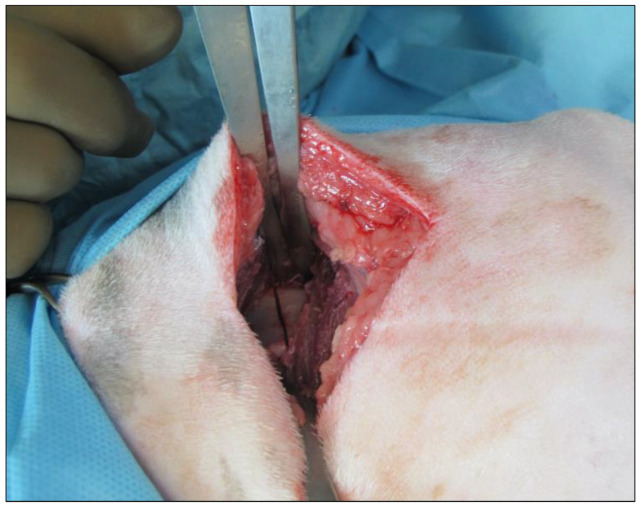
Intraoperative aspect of iliac osteotomy.

All cuts were safe and precise (Figure 2) without evidence of bleeding, intraoperative leg movement associated to the sciatic nerve involvement and adequate osteotomy lines.

The neurological assessment performed 6 and 24 h after surgery did not reveal any evidence of neurological disorders. All dogs were able to walk the day after surgery showing moderate weight-bearing lameness.

Neurological recheck including was then repeated at 14 days, 4 weeks and 8 weeks post-operatively. Evidence of no proprioceptive deficits, normal deep sensory perception upon squeezing digits, normal hock flexion on withdrawal were observed in all dogs (Table 1).

Further minor complications were included seroma in 7 out of 84 cases, 3 out of 84 superficial surgical site infections without involvement of the deep tissues. Single screws breakage was observed in three cases. No major complications were recorded.

**Figure 3:**
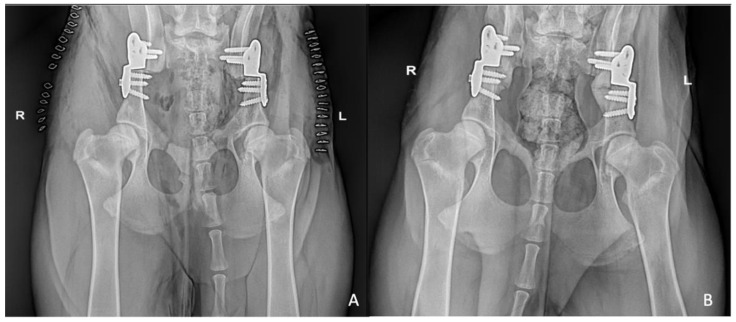
Immediate postoperative (**A**) and 8 weeks post-operative (**B**) VD standard hip radiographs of bilateral single stage DPO: pubic and iliac osteotomies were classified as grade 2.

Eight weeks after surgery 62 out of 84 pubic osteotomies were scored as grade 2, 22 out of 84 as grade 1. The iliac osteotomies were classified as grade 2 in all cases (Figure 3).

## 4. Discussion

Piezoelectric bone surgery is a technology based on the high frequency vibration of a metallic tip used to selectively cut bone while sparing surrounding soft tissues [23]. The technology is based on inverse piezoelectric activity: alternative current applied to piezoactive ceramic disks generates high-frequency vibratory energy [12,16,17].

In Veterinary Medicine Farrell and colleagues in an ex vivo study evaluated the efficacy of piezoelectric cutting tool for decompressive spinal surgery recording surgical duration and operating field visibility [12]. Results showed that the piezoelectric device allowed completion of ventral slots in a significantly shorter time, without an increased incidence of iatrogenic trauma [12].

In 2015, Hennet reported many uses for piezoelectric surgery in dentistry and in oromaxillofacial surgery, concluding that piezoelectric bone surgery constitutes a new and exciting field in veterinary oral and maxillofacial surgery [13].

In 2020, Crovace and collegaues reported the outcomes of 292 dogs and 32 cats which required spinal or skull osteotomy performed with the piezoelectric cutting tool because of a traumatic, degenerative or neoplastic lesion. The number of craniotomies and laminectomy/hemilaminectomy was 4 and 305, respectively. Two mechanical complications occurred attributable to the use of the piezoelectric bone scalpel (0.6%). They consisted in a dural tear and an epidural hematoma [21]. Histopathological evaluation of osteotomized surface showed the presence of live osteocytes and osteoblasts at the in 92% of cases suggesting that piezosurgery does not interfere with the healing process [21].

Following these encouraging results, we evaluated the safety of the piezosurgery in dogs underwent DPO procedures in which iatrogenic sciatic nerve injury may occur [1,8].

Iatrogenic damage of the sciatic nerve may be associated to different surgical procedures such as hip fractures, total hip replacement and double pelvic osteotomy [1,8,10].

In 2007, Forterre and colleagues reported the clinical features associated with iatrogenic sciatic nerve injury in dogs and cats admitted to their referral hospital [24]. Dogs were mainly involved, rather than cats. No breed predilection was observed but in most medium to large breed dogs (14 dogs) were more frequently affected which may reflect a more difficult surgical approach [24].

The double pelvic osteotomy was firstly described by Haudiquet and Guillon as triple pelvic osteotomy evolution in an ex vivo study [25]. According to Vezzoni DPO was associated to lower complication rates and better clinical outcome in comparison to TPO despite the DPO acetabular rotation was more difficult to performed [1]. In order to facilitate the acetabular iliac ventroversion the 2.5 pelvic osteotomy (2.5 PO) was described in an ex vivo study: pubic ostectomy and iliac osteotomy were followed by a dorsal monocortical ischial osteotomy. [3,6]. The sacrotuberous ligament and all soft tissues were removed to evaluate torsional stress on the bone after DPO and 2.5PO [3,6]. The sacrotuberous ligament may be part of the resistance to acetabular rotation; in the original TPO technique, Slocum and Devine suggested to release the sacrotuberous ligament [22]. In 2010, Vezzoni and colleagues released the sacrotuberous ligament in some cases to facilitate the rotation. However, with surgical experience, release of the sacrotuberous ligament was no more performed [1].

The iliac osteotomy together with the pubic ostectomy leads to the acetabular ventroversion [1,2,3,4,5,6,22,26]. Performing the iliac osteotomy, iatrogenic sciatic nerve damage may occur for both oscillating saw vibes and a direct accidental nerve involvement, screw insertion drilling and excessive retractors tension force. Following DPO sciatic nerve injury has been observed as consequence of an intraoperative occurred on the ischiatic notch fissure because of incomplete ilial osteotomy. and in a bilateral single-session DPO. In both dogs neuropraxia was no longer apparent 1 month postoperatively [8].

The sciatic nerve segments originating from the sixth and seventh lumbar nerves [27]. They fuse shortly after exiting the intervertebral foramina to become the larger lumbosacral trunk, which passes in the medial aspect of the ileal bone continues extrapelvically as the sciatic nerve. After crossing the greater sciatic notch, the nerve runs lateral to the femur. It is first seen dorsal to the quadriceps and then lateral to the adductor muscle, which is then followed by the semimembranosus muscle building the medial landmark. Proximal to the stifle the sciatic nerve terminates by branching into the common peroneal and tibial nerves.

In the present study the piezoelectric cutting tool allowed the iliac osteotomy without sciatic nerve damage. All the osteotomies were clean-cut and precise. No temporary and or permanent neurological disorders associated with the iliac osteotomy were recorded after surgery. In addition, the constant irrigation and the thin and sharp profile of the different instruments’ tips achieved the osteotomy site always free from blood and debris in all cases. In case of neurological disorders electrodiagnostic (electromyography and nerve conduction velocity) is recommended [24,28]. In case of irreversible sciatic nerve injury amputation of the affected pelvic limb needs to be considered. According to Hennet the main advantages associated with piezoelectric surgery have been: (1) Selective cut of mineralized tissue. Soft tissue vibrates without rupture at the same frequency as the tip of the instrument, significatively reducing trauma on vascular or neurological tissues. This aspect allows to achieve a safe osteotomy. Potentially soft tissues damage may occur when they are directly connected with the bone becoming unable to vibrate. (2) There was no pressure on bone avoiding thermal injuries and reducing bone microfractures. (3) The blood was drastically reduced with consequent reduction of post-operative complications such as edema, swelling and hematoma [13].

In the present cases no epidural and or local anesthesia were performed. The introduction of regional anesthesia (RA) in orthopedics surgery allowed to decrease general anesthetic requirements, stress response to surgery, provides postoperative analgesia in dogs [29,30]. We routinely performed loco-regional anesthesia in orthopedic surgery. Epidural anesthesia was not performed during DPO procedures to check any potential leg movement associated to the nerve involvement. Intraoperative and immediate post-operative pain management was achieved using intravenous opioids administration.

According to our results iliac osteotomy performed during double pelvic osteotomy may safe performed using the piezosurgery. A thorough case selection of DPO cases and a proper surgeon’s expertise is required to minimize any complications.

## Figures and Tables

**Table 1:** Dog Golden Retriever, F, 7 m: overview table of postoperative neurological (observation, palpation, postural reaction, spinal reflex and sensory evaluation), orthopedic (lameness) and radiographic assessment (pubic/iliac osteotomy healing). One example is given because of the similar findings.

Follow Up	Observation	Palpation	Postural Reaction	Withdrawal Reflex	Sensor Evaluation	Lameness	Pubic°/Iliac^•^ Osteotomy Healing
6 h po	n/a	n/a	n/a	n/a	n/a	moderate	
24 h po	n/a	n/a	n/a	n/a	n/a	moderate	
14 days po	n/a	n/a	n/a	n/a	n/a	mild	
4 wks po	n/a	n/a	n/a	n/a	n/a	slight	1°/1^•^
8 wks po	n/a	n/a	n/a	n/a	n/a	absent	2°/2^•^

PO = post-operative; h = hours; n/a = normal; wks = weeks. Osteotomy healing score: grade 0 = no signs of healing; grade 1 = incomplete osseous bridging; grade 2 = complete osseous bridging. Lameness was scored as absent, slight, mild, moderate and severe.

## Data Availability

The data generated in this study is already added in the tables of this article. If you need any further information, please feel free to contact authors.
